# Feasibility randomised controlled trial of Recovery-focused Cognitive Behavioural Therapy for Older Adults with bipolar disorder (RfCBT-OA): study protocol

**DOI:** 10.1136/bmjopen-2015-010590

**Published:** 2016-03-03

**Authors:** Elizabeth Tyler, Fiona Lobban, Chris Sutton, Colin Depp, Sheri Johnson, Ken Laidlaw, Steven H Jones

**Affiliations:** 1 Division of Health Research, The Spectrum Centre for Mental Health Research, Lancaster University, Lancaster, UK; 2 Lancashire Clinical Trials Unit, College of Health and Wellbeing, University of Central Lancashire, Preston, UK; 3 Department of Psychiatry, School of Medicine of the University of California, San Diego, San Diego, California, USA; 4 Department of Psychology, University of California, Berkeley, Berkeley, California, USA; 5 Faculty of Medicine and Health Sciences, Norwich Medical School, University of East Anglia, Norwich, UK

**Keywords:** Bipolar Disorder, Ageing, Randomised Controlled Trial, Cognitive Behavioural Therapy, Feasibility, Recovery

## Abstract

**Introduction:**

Bipolar disorder is a severe and chronic mental health problem that persists into older adulthood. The number of people living with this condition is set to rise as the UK experiences a rapid ageing of its population. To date, there has been very little research or service development with respect to psychological therapies for this group of people.

**Methods and analysis:**

A parallel two-arm randomised controlled trial comparing a 14-session, 6-month Recovery-focused Cognitive-Behavioural Therapy for Older Adults with bipolar disorder (RfCBT-OA) plus treatment as usual (TAU) versus TAU alone. Participants will be recruited in the North-West of England via primary and secondary mental health services and through self-referral. The primary objective of the study is to evaluate the feasibility and acceptability of RfCBT-OA; therefore, a formal power calculation is not appropriate. It has been estimated that randomising 25 participants per group will be sufficient to be able to reliably determine the primary feasibility outcomes (eg, recruitment and retention rates), in line with recommendations for sample sizes for feasibility/pilot trials. Participants in both arms will complete assessments at baseline and then every 3 months, over the 12-month follow-up period. We will gain an estimate of the likely effect size of RfCBT-OA on a range of clinical outcomes and estimate parameters needed to determine the appropriate sample size for a definitive, larger trial to evaluate the effectiveness and cost-effectiveness of RfCBT-OA. Data analysis is discussed further in the Analysis section in the main paper.

**Ethics and dissemination:**

This protocol was approved by the UK National Health Service (NHS) Ethics Committee process (REC ref: 15/NW/0330). The findings of the trial will be disseminated through peer-reviewed journals, national and international conference presentations and local, participating NHS trusts.

**Trial registration number:**

ISRCTN13875321; Pre-results.

**Strengths and limitations of this study**

- First randomised controlled trial to develop and test out a psychological intervention for older adults with bipolar disorder.
- Development of a psychological intervention for a group of people who currently have no evidence-based care.
- Recovery-focused Cognitive Behavioural Therapy for Older Adults with bipolar disorder (RfCBT-OA) has been developed in collaboration partnership with individuals with lived experience of bipolar disorder.
- RfCBT-OA has the potential to improve outcomes for service users. This would save the National Health Service (NHS) money through a reduction in use of mental health services.
- No active treatment control arm.

## Background

The UK population is ageing and this pattern is expected to continue into the next few decades.^1^ Current estimates suggest that approximately 10 million people in the UK are over 65 years old. The latest projections indicate that there will be 5½ million more older adults in the UK in 20 years’ time, and this number will have nearly doubled to 19 million by 2050.^2^ Consequently the number of older people living with chronic mental health problems is also set to rise substantially, including those with bipolar disorder (BD).^3^

There is limited research available on the presentation, course and treatment of BD in later life. Reasons cited for this lack of information include the increased mortality of younger individuals with BD, sampling biases in the research studies that are available, changes in the diagnostic criteria over time and differences in research settings where individuals are studied.^4^

Available data indicate that rather than early theory suggestions that BD ‘burns out’,^5^ the majority of individuals that experience early onset BD will follow a chronic and relapsing course into older adulthood.^6^ Older adults with BD may face additional challenges such as cognitive impairments^7^ and a decline in health-related quality of life.^8^ ^9^ BD in later life is also associated with a high risk of suicide^10^ and significant service costs. Bartels *et al*^11^ reports that older adults with BD utilise almost four times the total use of mental health services and are four times more likely to be hospitalised and get admitted than older people with unipolar depression.

Despite this evidence of the importance of BD in older adults, there has been very little research or service development for this group particularly with respect to psychological therapies.^12^ The National Institute for Health and Care Excellence (NICE) BD guideline^13^ recommends that older adults should be offered the same treatment as younger people. However, there are no published studies evaluating psychosocial interventions for older adults with BD,^14^ and a number of reviews have highlighted the relative paucity of knowledge concerning our knowledge in this area.^4^ ^15^ ^16^

Although research into psychological therapies for older adults with BD is lacking, there is evidence for the effectiveness of such interventions in adults of working age.^3^ ^17^ ^18^ Although recovery-informed interventions are now recommended by the UK government,^19^ ^20^ much of the available research to date has focused on cognitive-behavioural therapy (CBT) and psychoeducational approaches designed to reduce relapse risk but with little explicit focus on functional outcomes including personal recovery. There is no single definition of ‘recovery’ in mental health. However, it is based on the principle that it is possible for an individual to gain a meaningful life, while living with a serious mental health problem. Unlike recovery from a physical illness, in mental health, the person may aim for recovery, while still experiencing some of the symptoms of their problem. There is an emphasis on having a set of goals which may focus on re-establishing other areas in a person's life such as their work, relationships or social life.

A recent randomised controlled trial (RCT) study has shown that a recovery-focused CBT intervention (Recovery-focused Cognitive Behavioural Therapy, RfCBT) for individuals with BD (below 65 years) is beneficial in terms of both functional and symptomatic outcomes.^21^ The present trial builds on this work and has adapted RfCBT, so that it specifically meets the needs of an older adult population (RfCBT-OA). Details of how these adaptations were achieved can be found in the intervention section.

We therefore intend to perform a RCT to evaluate the effectiveness and cost-effectiveness of RfCBT-OA plus treatment as usual (TAU) compared with TAU. However, there are a number of uncertainties that we need to address prior to initiating that trial. Therefore, in this feasibility study, we plan to evaluate the feasibility and acceptability of the RfCBT-OA intervention and whether a full RCT is feasible. We will evaluate recruitment into the study (both self-referral and clinician referral), consent to participate and participant attrition rates (overall and each study arm separately) during assessment, intervention and follow-up periods and completion of outcome measures. We will also be measuring adherence to the intervention (number of therapy sessions attended, therapy drop out and feedback from qualitative interviews at the end of therapy). This will allow us to evaluate the acceptability of the intervention to the individuals taking part in the study. The trial will also provide initial data on the potential impact of the intervention (compared with current routine care) on a number of clinical outcomes and help to identify the most appropriate primary outcome (eg, perceived recovery, time to relapse and mood symptoms) for a definitive clinical RCT in the future.

## Methods

This protocol is guided by the Standard Protocol Items: Recommendations for Intervention Trials (SPIRIT) 2013 Guidelines.^22^ The study is registered with the ISRCTN registry: ISRCTN13875321. A model consent form is provided in online supplementary appendix 1.

10.1136/bmjopen-2015-010590.supp1 Supplementary appendix

### Objectives

To determine the feasibility and acceptability of a recovery-focused CBT intervention for older adults with BD compared with TAU.

The objectives of the study are to:

1. Investigate
  - Whether clinicians working with older adults will refer participants into a RCT;
  - Whether older adults will self-refer into a RCT;
  - Whether older adults with BD will consent to participate in a RCT of a psychological intervention;
  - Participant attrition rates (overall and each study arm separately) during assessment, intervention and follow-up periods;
2. Determine the acceptability of the recovery-focused intervention in terms of
  - Whether individuals adhere to and engage with the intervention;
  - Participants’ experiences of the intervention;
3. Identify the most appropriate primary outcome measure (eg, recovery, time to relapse, quality of life) for a future trial;
4. Estimate parameters needed to determine the appropriate sample size for a future trial to evaluate the effectiveness and cost-effectiveness of RfCBT-OA.

### Trial design

**Figure 1:**
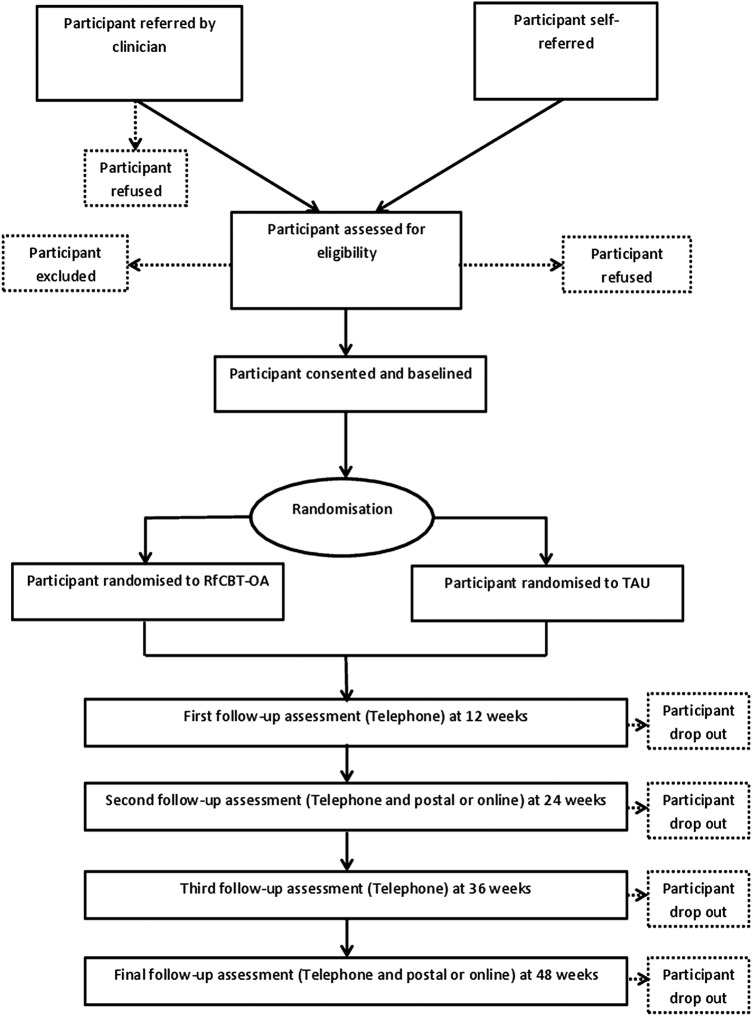
Flow diagram showing design of the study. RfCBT-OA, Recovery-focused Cognitive Behavioural Therapy for Older Adults with bipolar disorder; TAU, treatment as usual.

A parallel, two-arm RCT comparing a 14-session, 6-month RfCBT-OA intervention alongside TAU versus TAU alone. Participants in both arms of the study will complete assessments which will include a range of important clinical outcomes (eg, recovery, time to relapse, quality of life) at baseline and then three monthly over the 12 month follow-up period (see figure 1). Rater blindness will be achieved by having an independent researcher from the Spectrum Centre team as ET will deliver the intervention.

A trial steering committee (TSC) will be formed at the beginning of the trial. It will consist of an independent chair, independent clinician (s), an independent statistician, a service user representative and the researcher. They will meet face to face on four occasions over the duration of the trial. The TSC will concentrate on progress of the trial, adherence to the protocol, and importantly the rights, safety and well-being of the trial participants. TSC will review any adverse events should these occur and will advise on adaptation or termination of the intervention should this be required.

### Sample

#### Sample size

A formal power calculation is not appropriate as the primary purpose of the study is to evaluate the feasibility and acceptability of delivering the proposed intervention. It has been estimated that randomising 25 participants per group will be sufficient to be able to reliably determine the primary feasibility outcomes. The recruitment target has been set at 50 participants in line with recommendations for sample sizes for feasibility/pilot trials^23^ and to allow for expected attrition rates (see table 1). This number will also allow us to evaluate the other objectives of the trial; to assess the impact of the intervention on each of the outcome measures; to estimate parameter necessary to design a main trial; and will enable estimation of recruitment and retention parameters with sufficient precision. For example, recruiting 50 participants will enable estimation of the percentage attrition to within ±10% if attrition is 15% or less and, if the consent rate is 80%, approaching 63 participants and recruiting 50 will enable estimation of the consent rate to within ±10%.

**Table 1:** Feasibility outcomes thresholds

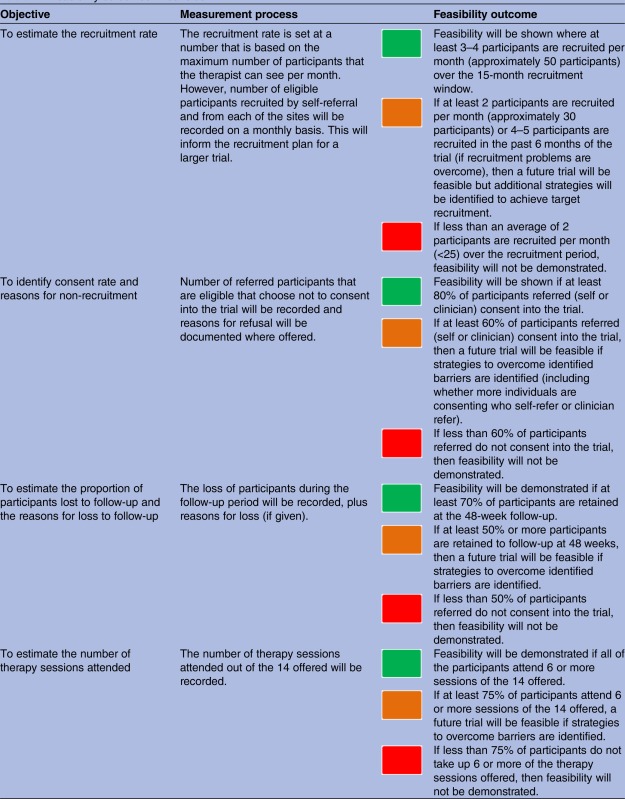
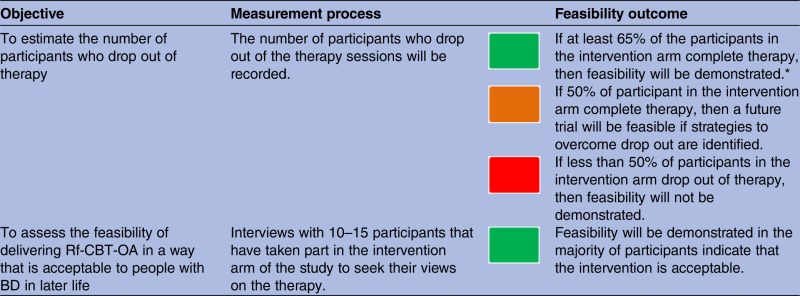

Red—stop—main study not feasible.

Amber—continue but modify protocol—feasible with modifications.

Green—continue without modifications—feasible as is.

*Based on the percentage of drop out of older adults (33–37%) in comparable studies investigating psychotherapeutic treatment for depression in later life^36–39^ psychotherapeutic treatment trials.

BD, bipolar disorder; RfCBT-OA, Recovery-focused Cognitive Behavioural Therapy for Older Adults with BD.

#### Inclusion/exclusion criteria

1. A diagnosis of BD (I or II) according to the Structured Clinical Interview for the Diagnostic and Statistical Manual of Mental Disorders (SCID)^24^ IV research criteria;
2. Not in a current episode of mania, hypomania, depression or mixed episode in the last month;
3. Aged 65 or above;
4. Sufficient English language skills to comprehend the assessments and intervention content.

Exclusion criteria:

1. Receiving concurrent psychological therapy;
2. A score of less than 22 on the Montreal Cognitive Assessment (MoCA).^25^

#### Recruitment

Referrals will be sought from participating National Health Service (NHS) Trusts in the North-West, UK, with support from the National Institute of Mental Health (NIHR) Clinical Research Network. This is a publicly funded national workforce that supports the recruitment of participants to nationally funded research studies. The lead researcher will contact managers in older adult community mental health teams, outpatient clinics, general practitioner (GP) surgeries and primary care mental health teams. They will request to attend any planned team meetings and send the participant information sheet and referral information for distribution within the team. The researcher will follow-up any visits with a phone call and ask the health professional (eg, psychiatrist, GP, care coordinators) to complete the referral information sheet and send it via email or post to the research team. All referrals received will be recorded on a confidential database and participants will then be approached to book a screening interview. The researcher will also visit service user groups (such as Bipolar UK, MIND and Rethink) in the local area. The researcher will take the study self-referral form and ask any interested participants to either complete the form in the group or send it back to the research team by post or email. Any self-referrals received will be recorded on a confidential database. The study will also be advertised through a well-established, confidential volunteering database at the Spectrum Centre which has contact details for over 500 individuals that have either lived experience or an interest in BD as well as through social media such as Facebook and Twitter and in the media. Posters and leaflets will be distributed in both NHS and non-NHS sites to maximise participant access.

### Screening, baseline and randomisation

Once participants have expressed an interest in participating, they will be contacted by a member of the research team to complete a brief screening interview. The screening interview will be conducted over the telephone and will consist of questions targeting inclusion and exclusion criteria. The Mood Disorder Questionnaire (MDQ)^26^ will also be administered. This is a brief self-report screening instrument that identifies individuals likely to have BD. At this stage, all participants will be asked to provide consent for the researcher to contact a nominated healthcare professional to obtain risk-related information (eg, GP, care coordinator). Individuals who meet both the eligibility criteria and screen likely on the MDQ^26^ will then be booked in for an initial assessment.

Assessments will take place at the Spectrum Centre or the participant's home, according to preference. If required, private space for appointments may also be negotiated at willing primary, secondary and/or voluntary organisations. At the initial visit, the study will be described to potential participants in full. The voluntary nature of participation will be emphasised, including the right to withdraw at any time.

Information collected during the initial assessment will be used to confirm eligibility. Once informed consent is obtained, the baseline assessment will be conducted by the researcher. The MoCA^25^ will be used as the first screening tool for eligibility as it is the least time consuming. The MoCA^25^ assesses for cognitive impairment via multiple cognitive domains including attention, concentration, executive functions, memory, language, visuospatial skills, abstraction, calculation and orientation. If the participant scores 22 or above on the MoCA,^25^ then the SCID^24^ interview will be carried out to confirm a diagnosis of BD. This will also identify whether they have had an episode in the past month, to provide demographic information and assess the number of previous episodes. Individuals who score below 22 on the MoCA^25^ or do not meet the research criteria for BD will be thanked for their time and informed that they do not meet the research criteria required to participate. They will be offered the option of joining Spectrum Connect (our participant panel), so that they can find out about future research/activities that may be of greater relevance. If the participant meets criteria and wishes to take part, they will complete the baseline clinical outcome measures that are detailed below.

After baseline, the participant will be randomly allocated to either RfCBT-OA or TAU using an independent web-based computer-generated randomised procedure (http://www.sealedenvelope.com/) to aid allocation concealment. The randomisation process will be set up by Lancashire Clinical Trials Unit (CTU). After randomisation, the researcher will contact the nominated healthcare professional to inform them that the participant is taking part in the study. Intentional unblinding will only be allowed if necessary for patient safety; any unintentional unblinding will be recorded (including reason) and subsequent assessments conducted by another blind researcher. Data entry procedures and storage will be overseen by the CTU. All personal information will be securely stored in line with NHS ethical approval.

### The recovery-focused CBT intervention

The original RfCBT manual was developed from key components of effective CBT interventions which include mood monitoring and awareness, regularisation of routines, enhancing prodromal coping and problem-solving training^27–31^ and refined by qualitative research to capture experiences of recovery in BD and through service user focus groups to ensure that the content, focus and delivery of the intervention was in tune with service user recovery priorities. The intervention places emphasis on maintaining a very flexible engagement approach with respect to initial rapport building and consideration of timing, duration and frequency of sessions. It is focused on helping individuals work towards goals that are of personal value to them, whether symptom related or about other areas of their lives such as work or social support. Initially, the client and therapist develop a shared understanding of recovery and how working towards the recovery goals may have a significant impact on the individual's life. The intervention includes a significant formulation component, ensuring that any therapeutic approaches are consistent with the client's current needs.

Recovery-focused therapy^32^ has the following phases:

1. Introducing the recovery approach to clients;
2. Collection of information about current and historical mood and functioning;
3. Meaning and relevance of diagnosis;
4. Identification of recovery-informed therapy goals;
5. Initial formulation of relationships between mood experiences and progress towards recovery goals;
6. Identification and application of CBT techniques to address and facilitate positive coping;
7. Consideration of wider functioning issues in relation to recovery;
8. Development and completion of early warning signs (EWS) plan;
9. Development and completion of recovery plan;
10. Sharing lessons from therapy with key stakeholders.

Although it is likely that most clients will engage with most of these elements, the relative emphasis will depend on the individual goals and formulation of the particular client. An additional chapter has been developed for the manual, so that it specifically meets the needs of an older adult population. This has been achieved by a review of current evidence for adapting psychological interventions for older adults with mental health problems. There has also been extensive consultation (focus groups and one-to-one) with service users with lived experience of BD in later life, their relatives and experts in the field.

Data from the focus group have identified that individuals living with BD in later life still experience episodes, however to varying degrees. Some find their episodes are more manageable and the symptoms are less intense, some feel that they are worse than when they were younger and are harder to control. Individuals taking part in the focus group felt that therapy in later life should focus on psychoeducation, symptom management and also to consider wider areas of functioning such as achieving meaningful activity. This fits with the flexible, idiosyncratic approach that the recovery-focused therapy offers. The older adults identified additional difficulties in later life such as physical health problems, memory difficulties key themes such as loneliness, losses and changes in role. These correspond with the current literature on adapting psychological therapies for older adults.^33^ ^34^

Therefore, key areas for adaptation in the new chapter focus on memory and learning, physical health difficulties and sensory impairments. A number of age-related themes such as cohort beliefs, role investments, intergenerational linkages and the sociocultural context^34^ are also discussed as potential areas of adaptation.

### Outcomes

#### Feasibility and acceptability data

To address the primary objective and allow the evaluation of the feasibility and acceptability of delivering the recovery-focused CBT intervention to older adults with BD, a number of outcomes will be assessed. Setting benchmarks for feasibility data will be beneficial to inform a larger scale assessment of this intervention in the future.^35^

Detailed information will be collected which will include the number of referrals received per month, the source of recruitment (health professional vs self-referrals), number of participants contacted, assessed for eligibility and consented into the trial. Reasons for non-eligibility or withdrawal of interest will be documented, where given. Retention of participants in both arms of the trial will be assessed during assessment, intervention and follow-up periods, and the completion of outcome measures. Feasibility outcomes will be measured using detailed thresholds and a traffic light system described in table 1 below.

To determine the acceptability of the intervention, quantitative data (eg, number of sessions attended and drop outs) will be combined with data from a set of qualitative interviews. These will be conducted to explore individuals’ experiences of receiving RfCBT-OA intervention in more detail. A sample (approximately n=10–15) will take part in a topic-guided qualitative interview. The sample will be selected purposively across key characteristics (eg, age, gender, attendance rates) to create a diverse sample of people which will include people who completed the intervention and also people who dropped out. It was felt that this number will provide sufficient data to provide additional information for the feasibility outcomes and the acceptability of the intervention. The interviews will also identify issues and strategies necessary to inform the design of a larger trial in the future.

The qualitative data plus the feasibility trial data will help to allow us to achieve the other objectives of the study which are to identify the most appropriate primary outcome measure and to estimate parameters needed to determine the appropriate sample size for a future trial. Further details can be found in the Analysis section.

#### Clinical outcome data

The SCID^24^ and MoCA^25^ will be completed at baseline to confirm the participants’ bipolar diagnosis. The follow-up period will be 12 months from initial randomisation. There will be regular 3-month assessments to evaluate bipolar relapse, bipolar symptoms and functioning over the telephone. In addition to this, an assessment of recovery, mood symptoms and quality of life will be completed at baseline, end of therapy and follow-up (6 and 12 months). These self-report measures will be completed either by post or online using participant preference. See table 2 for assessment measure schedule.

**Table 2:** Assessment schedule

		Follow-up period (weeks)					
Measure	Baseline face to face	12 Phone	24 Phone	Postal/online	36 Phone	48 Phone	Postal/online
SCID	*						
MoCA	*						
SCID-LIFE	*	*	*		*	*	
HDRS	*	*	*		*	*	
MRS	*	*	*		*	*	
PSP	*	*	*		*	*	
BRQ	*			*			*
ISS	*			*			*
CES-D	*			*			*
WSAS	*			*			*
WHOQoL-Bref	*			*			*
QoL.BD	*			*			*

CES-D, Centre for Epidemiologic Studies Depression Scale; HDRS, Hamilton Depression Rating Scale; ISS, Internal State Scale; MoCA, Montreal Cognitive Assessment; MRS, Mania Rating Scale; PSP, Personal and Social Performance Scale; QoL.BD, Quality Of Life in Bipolar Disorder Scale; SCID, Statistical Manual of Mental Disorders; WHOQoL-Bref, WHO Quality of Life Scale; WSAS, Work and Social Adjustment Scale.

#### Interviewer-rated measures

*The Structured Clinical Interview for Diagnosis: Research Version*^40^ provides longitudinal information on Diagnostic and Statistical Manual of Mental Disorders, Fourth Edition (DSM-IV) episodes (major depression, mania, hypomania or mixed affective episode). It includes items from the SCID as well as the Hamilton Depression Rating Scale (HDRS)^41^ and Mania Rating Scale (MRS).^42^ The SCID-LIFE will be delivered every 3 months over the telephone following baseline to generate weekly scores of mania and depression on a 1–6 severity scale. Scores of 5/6 indicate presence of symptoms and impact on functioning that corresponds to symptom criteria for major mood episode as defined by the DSM-IV. Weekly scores will be used to examine the number of weeks out of episode (a score of 4 or less on SCID-LIFE), number of weeks without impairment (a score of 2 or less on SCID-LIFE) and time to first episode of depression and mania.

*Personal and Social Performance Scale*^43^: The Personal and Social Performance Scale (PSP) is an interview schedule to assess functioning in the domains of socially useful activities, personal and social relationships, self-care, and disturbing and aggressive behaviours. Good inter-rater reliability has been reported.^43^ It has been used previously to assess outcome in response to treatment for BD.^22^

#### Self-report outcome measures

*The Bipolar Recovery Questionnaire (BRQ*)^44^ is a self-report measure designed to assess personal experiences of recovery in BD. The BRQ is scored out of 3600 (a higher score indicates a higher degree of self-rated recovery). The BRQ is internally consistent and reliable over test-retest period.^44^ There is also evidence that the BRQ is sensitive to change in a recovery-focused CBT trial for early BD.^22^

*The Internal State Scale (ISS)*^45^ is a 15-item self-report measure that assesses symptoms of mania and depression. It compromises of four subscales: activation, perceived conflict, well-being and depression. Each statement is rated based on how the individual has felt in the past 24 h. A cut-off score of >200 on activation scale has been validated as indicative of the presence of (hypo)mania when accompanied by a score of >125 on the well-being scale.^45^

*The Centre for Epidemiologic Studies Depression Scale (CES-D)*^46^ is a 20-item self-administered scale designed to measure depressive symptoms in the general population. The scale measures the major components of depressive symptomatology, including depressive mood, feelings of guilt and worthlessness, psychomotor retardation, loss of appetite, and sleep disturbance. Each item is scored on a four-point Likert scale to determine a level of severity score: <15 (no depression); 15–21 (mild-to-moderate depression); >21 (possibility of major depression).

*Work and Social Adjustment Scale (WSAS)*^47^ is a brief five-item measure of functioning in the domains of work, home management, social leisure, private leisure and relationships. There is a maximum score of 40 (a higher score indicates higher severity of difficulties). It has been extensively used in longitudinal research on BD.^48^ ^49^

*The WHO Quality of Life Scale (WHOQOL-Bref)*^50^ comprises of 26 items, which measure the following broad domains: physical health, psychological health, social relationships and environment. The scores from the four domains are transformed on a scale from 0 to 100.

*Quality Of Life in Bipolar Disorder Scale (QoL.BD)*^51^ is a 12-item disorder-specific questionnaire used to assess quality of life in BD within several areas including physical, sleep, mood, leisure, spirituality and identity. The QoL.BD is scored out of 60 (a higher score indicates higher perceived quality of life). Initial field testing of the quality of life in BD supports use of the instrument as a feasible, reliable and valid disorder-specific quality of life measure for BD.^51^

#### Measures to assess therapeutic alliance

*The Working Alliance Inventory—Short form, therapist and client version (WAI-S)*^52^ is a 12-item questionnaire that measures the strength of the therapeutic alliance between both therapist and client. The WAI-S measures three dimensions of alliance: bond, goals and tasks. Two versions of the WAI-S will be used; one specific for the client, and one for the therapist, both of which will be administered twice across the 14 therapy sessions. The WAI-S has received psychometric support, has good overall internal consistency (α=0.94), and good internal consistency for each dimension of alliance, including bond (α=0.84), goals (α=0.88) and tasks (α=0.90).

## Analysis

### Feasibility

The key focus of the trial is on issues of feasibility and acceptability of the intervention. Much of the analysis will therefore be based around summary statistics used to estimate key parameters: rates of recruitment, demographics of sample, and retention to therapy and follow-up assessments. These summary statistics will be accompanied by 95% CIs.

### Clinical outcomes

In line with recommendations for sample sizes for feasibility/pilot trials,^5^ obtaining outcome data from at least 80%^20^ participants per group will be sufficient to address key objectives (such as the estimation of the SD of a quantitative outcome or the proportion with a dichotomous outcome) with adequate precision.

Generalised linear mixed models will be used to assess the impact of the intervention on each of the continuous outcome measures to estimate parameters necessary to design the main trial. Time to first relapse will be analysed using time-to-event methods, including Kaplan-Meier estimation and the Cox proportional hazards regression model. Separate analyses will be performed for the three different types of recurrence (any, depressive and hypomanic/manic episodes). Analyses will be conducted on an intention-to-treat basis and key parameter estimates will be presented as point estimates with 95% CIs.

A number of factors will be analysed to help identify a primary clinical outcome for a main trial (eg, recovery, time to relapse and mood symptoms). Each measure will be assessed in relation to its sensitivity to change, completion rates and acceptability which will be explored further during the qualitative interviews.

### Qualitative data

Data from qualitative interviews will be analysed using a process called thematic analysis^53^ ^54^ which focuses on examining themes in the data and identifying implicit and explicit ideas. The qualitative transcripts will be read and coded using a coding frame that will be developed as the data analysis progresses. The codes will be organised into thematic headings and the data will be reordered and summarised into themes. The analysis will be crosschecked by another member of the research team to ensure validity.

### Dissemination plans

The team intend to publish the outcomes from the trial in peer-reviewed journals but will also try to reach public audiences including people living with BD through third sector events and contributions to third section publications as well as use of social media. No professional writers will be used and all authors will contribute substantively to final manuscripts.

## Discussion

This study aims to develop and test the feasibility and acceptability of the RfCBT-OA intervention for older people living with BD. The data from the trial will allow us to determine rates of recruitment and retention and identify factors which may help improve these rates if a future trial is feasible. The acceptability of the therapeutic intervention will be assessed by evaluating the therapy attendance rates, drop outs and feedback from the qualitative interviews. We will also be able to gain an estimate of the likely effect size of RfCBT-OA on a range of clinical outcomes. All of these data are essential to inform the design of a large-scale trial. Detailed feasibility outcome thresholds have been set in table 1. These will need to be met in order to progress to a further, definitive evaluation trial of the clinical and cost-effectiveness of RfCBT-OA.

The original recovery intervention, RfCBT,^32^ was developed in collaboration partnership with individuals with lived experience of BD. This included service user involvement in qualitative work on recovery experiences and a structure and format of the RfCBT intervention. As highlighted by Jones *et al*,^32^ engaging individuals with personal experience of BD at this level fits with the model of recovery approaches as being empowering, individualised and grounded in the individual's own priorities and needs. The current RfCBT-OA intervention has been further refined by a group of older adults living with BD and experts in the ageing field.

Strengths of the study include the development of an intervention for a group of people who currently have no evidence-based care. The Department of Health^55^ states that older adults with mental health problems should have access to the same range of therapies as those people under the age of 65. This is not the case for people with BD. There are currently no published studies evaluating psychological interventions for older adults with BD and there is a clear need to develop an evidence base for this population.

The rapid ageing of the population will make significant demands on healthcare services, especially if the current lack of evidence-based treatments continues. The development of a recovery-focused psychological intervention has the potential to improve outcomes for service users, helping them to develop a range of coping strategies and putting them more in control of managing their mental health problems. This would save the NHS money through a reduction in use of mental health services. The intervention also offers a flexibility to work on a range of outcomes. Focus group work with this population has identified that individuals are still experiencing episodes in later life and want the flexibility to work on both symptom management and other areas of their life. The recruitment for the study will take place across primary and secondary mental health services and through self-referral. There is the hope that not restricting recruitment to specialist mental health services will allow a more representative sample.

There are a number of limitations to the study. First, there is no active treatment control arm, so any indications that the intervention is effective may not be specifically related to the recovery-focused intervention per se. Second, the scale of the study allows a follow-up period of only 6 months following therapy. A longer follow-up period might have been more helpful to assess the impact of the intervention and whether individuals would complete assessment measures over a longer time period. However, the primary aim of the study is to assess the feasibility and acceptability of the intervention; therefore, the 6-month follow-up window is a first appropriate step to help to assess whether a further, definitive RCT is feasible in the future. Third, as this is the first intervention study for older adults with BD, there are no well-validated measures for this population. The bipolar-related measures have not yet been specifically validated for use with an older adult population. However, the samples for the development papers for the BRQ^44^ and the QoL.BD^51^ included people over the age of 60. Additionally, focus group data indicate that outcomes such as personal recovery and quality of life are still important over the age of 60.

Despite these limitations, if this intervention is feasible to deliver, it offers a promising step for a group of people that currently does not have access to evidence-based psychological care.
