# Supplementary material for: Feasibility randomised controlled trial of Recovery-focused Cognitive Behavioural Therapy for Older Adults with bipolar disorder (RfCBT-OA): study protocol
Source: BMJ Open. 2016 Mar 3;6(3):e010590. doi: 10.1136/bmjopen-2015-010590 (PMC4785318; doi:10.1136/bmjopen-2015-010590)
Supplement: Supplementary appendix [file bmjopen-2015-010590supp_appendix.pdf]

Lancashire Care NHS
NHS Foundation Trust

Health Research | Lancaster University

APPENDIX 1- MODEL CONSENT FORM

Recovery Focused Therapy for Older Adults with Bipolar Disorder

REC ref:

Name of Researcher: ____

Name of Participant: ____

Participant Number ☐☐☐☐☐☐

PART ONE

Please initial box

1. I confirm that I have read and understood the information sheet version number 1.0 dated 12/02/2015 for the above study and have had the opportunity to ask questions. ☐

2. I understand that my participation is voluntary and that I am free to withdraw at any time, without giving any reason, and without my medical care or legal rights being affected. I understand that should I withdraw then the information collected so far cannot be erased and that this information may still be used in the analysis and publication of this study. ☐

3. I give my consent for the research team to contact my care co-ordinator and / or GP / other health professional in order to obtain risk-related information. ☐

4. I agree to my GP (and care co-ordinator where appropriate) being informed of my participation in this study and being informed should the research team be concerned about my mental health whilst taking part in this study. ☐

5. I agree to being contacted by the research team for a maximum of 12 months to complete interview and questionnaire assessments, in person, over the telephone and on-line, to find out how I am. ☐

6. I understand that my medical notes and records may be made available to responsible individuals from Lancaster University, your relevant North West NHS trust the research group and regulatory authorities where it is relevant to my taking part in this study. I give permission for these individuals to have access to my records and to collect, store, analyse and publish information obtained from my participation in this study. I understand that my personal details will be kept confidential and all data published will be anonymous. ☐

7. I agree to take part in the above study. ☐

______________________	______________	____________________
Name of Participant	Date	Signature
______________________	______________	____________________
Name of Person taking consent<br>(If different from Principal Investigator)	Date	Signature
______________________	______________	____________________
Name of Principal Investigator	Date	Signature

THE SPECTRUM CENTRE

THE SPECTRUM CENTRE

shire Care NHS
oundation Trust

Health Research | Lancaster University

PART TWO

---

1. I give my consent for my assessment interviews (initial, baseline and follow-up) with the researchers, both in person and over the telephone, and sessions with the therapist to be audio-taped. I understand that this is so the research team can ensure that the information I have provided has been documented accurately, to confirm that the researchers are using assessment and therapy materials consistently and for the purposes of supervision. *Declining to do so will not affect my participation in the trial in any way.* ☐

2. I give my consent for my direct, anonymised quotations to be recorded and used if required in the appropriate published format. *Declining to do so will not affect my participation in the trial in any way.* ☐

3. I would/would not (please delete as appropriate) like the results of my initial and/ or follow-up assessment(s) to be shared with my care co-ordinator / GP / other professional involved in my care. *Declining to do so will not affect my participation in the trial in any way.* ☐

4. I would like to be informed of the results of this study. *Declining to do so will not affect my participation in the trial in any way* ☐

5. ***Optional:*** If I am allocated to the treatment arm of the study I agree to be contacted about taking part in an interview about my experiences of receiving recovery focused CBT. *Declining to do so will not affect my participation in the trial in any way* ☐

6. ***Optional:*** If I am allocated to the treatment arm of the study and later choose to opt out of the study, I agree to be contacted about taking part in an interview about why I no longer want to take part. *Declining to do so will not affect my participation in the trial in any way* ☐

________________________	_______________	______________________
Name of Participant	Date	Signature
__________________________	_______________	______________________
Name of Person taking consent<br>(If different from Principal Investigator)	Date	Signature
__________________________	_______________	______________________
Name of Principal Investigator	Date	Signature

3 copies: 1 for participant, 1 for the project notes and 1 for the medical notes

THE SPECTRUM CENTRE
